# Wake up, Connecticut!

**Published:** 1897-04

**Authors:** 


### WAKE UP, CONNECTICUT!

WHAT is the matter with the Nutmeg State Veterinary Association? Why is it not more actively in the harness? Surely the load has not been all drawn up the hill there, and the descent of ease secured. Surely they have not all the laws in that State that are needed for the protection of their people from charlatans and from contagious and infectious diseases. Surely the millennium of meat- and milk-inspection has not been reached. We have not heard of any quarantine regulations to guard the borders of their State. There must be danger in all these directions which must be provided against. And this work is the duty of every State organization. The Nutmeg State has a large number of good men, intelligent veterinarians and interested professional men, and a shoulder-to-shoulder struggle will secure all the desired results. Sister Associations are ready to help. The JOURNAL is yours for support, information, and a helping hand. Wake up, colleagues, and command us all to help you!

